# Somatomotor Disconnection Links Sleep Duration With Socioeconomic Context, Screen Time, Cognition, and Psychopathology

**DOI:** 10.1016/j.bpsgos.2025.100522

**Published:** 2025-04-30

**Authors:** Cleanthis Michael, Aman Taxali, Mike Angstadt, Katherine L. McCurry, Alexander Weigard, Omid Kardan, M. Fiona Molloy, Katherine Toda-Thorne, Lily Burchell, Maria Dziubinski, Jason Choi, Melanie Vandersluis, Luke W. Hyde, Mary M. Heitzeg, Chandra Sripada

**Affiliations:** a Department of Psychology, University of Michigan, Ann Arbor, Michigan; b Department of Psychiatry, University of Michigan, Ann Arbor, Michigan; c Survey Research Center at the Institute for Social Research, University of Michigan, Ann Arbor, Michigan

**Keywords:** Brain development, Graph theory, Multivariate predictive modeling, Risk and resilience, Sleep duration, Somatomotor disconnection

## Abstract

**Background:**

Sleep is critical for healthy brain development and emotional well-being, especially during adolescence, when sleep, behavior, and neurobiology are rapidly evolving. Theoretical reviews and empirical research have historically focused on how sleep influences mental health through its impact on higher-order brain systems. No studies have leveraged data-driven network neuroscience methods to uncover interpretable, brainwide signatures of sleep duration in adolescence, their socioenvironmental origins, and their consequences for cognition and psychopathology.

**Methods:**

We implemented graph theory and component-based predictive modeling to examine how a multimodal index of sleep duration (parent-report, youth-report, Fitbit) is associated with intrinsic brain architecture in 3037 youths (ages 11–12 years) from the ABCD (Adolescent Brain Cognitive Development) Study.

**Results:**

We demonstrated that network integration/segregation exhibited a strong, generalizable multivariate association with sleep duration (*r* = 0.23, *p* < .001). The multivariate signature of shorter sleep predominantly involved increasing disconnection of a lower-order system, the somatomotor network, from other systems. Next, we identified a single component of brain architecture as the dominant contributor of this relationship (*r* = 0.15), which again exhibited this somatomotor disconnection motif. Finally, greater somatomotor disconnection was associated with lower socioeconomic resources, longer screen times, reduced cognitive/academic performance, and elevated externalizing problems (βs > 0.03, *p*s ≤ .007).

**Conclusions:**

These findings reveal a novel neural signature of shorter sleep in adolescence that is intertwined with environmental risk, cognition, and psychopathology. By robustly elucidating the key involvement of an understudied brain system in sleep, this study can inform theoretical and translational research directions on sleep to promote neurobehavioral development and mental health during the adolescent transition.

## Plain Language Summary

In this study, we sought to uncover brainwide signatures of shorter sleep duration during adolescence, a period often characterized by inadequate sleep. Whereas the dominant perspective is that sleep primarily influences cognitive brain systems, our findings demonstrate that shorter sleep duration is predominantly associated with the somatomotor system, which underlies motor control. In adolescents who sleep less, the somatomotor system is more disconnected from the rest of the brain. Somatomotor disconnection is also related to relevant risk factors (socioeconomic resources, screen time) and outcomes (cognition, psychopathology). These findings challenge our current understanding of how sleep influences health, brain function, and development.

Sleep is a basic need and plays a critical role in cognition, emotion regulation, academic and occupational attainment, and physical and mental health (1). Experimental, longitudinal, genetically informed, and clinical trial designs have demonstrated that sleep causally influences health and behavior across the lifespan (2, 3, 4, 5). However, most adolescents sleep less than recommended cross-culturally (6,7). Given systemic inequities, inadequate sleep during adolescence is more common within disadvantaged communities (8), perpetuating population-level health disparities. Similarly, most psychiatric disorders emerge in adolescence (9), partially due to changing trends in sleep (10). Accordingly, the National Sleep Foundation has declared adolescent sleep deprivation a public health epidemic (11). Numerous factors unique to adolescence may shorten sleep such as increasing academic and social demands, longer screen times, circadian and physiological remodeling, and early school start times (12). These considerations underscore the importance of delineating the biological mechanisms through which sleep duration influences health and behavior during adolescence, which can inform risk identification, policy, and intervention.

Cross-species evidence demonstrates that a central function of sleep is to promote healthy brain development (13). Adolescent sleep patterns have been associated with the structure and function of individual brain regions (12). Most research has adopted a theoretically driven approach, examining an a priori subset of regions that underlie higher-order processes including executive functions, emotion regulation, decision making, learning, and memory (e.g., prefrontal-limbic regions) (14). This literature has begun to illuminate how normative and extreme variations in adolescent sleep patterns become neurobiologically and behaviorally expressed.

Because cognition, emotion, and psychiatric risk emerge from coordinated activity across the entire brain (15,16), focusing on specific regions may not capture how the entire brain is impacted by sleep to shape health and development. Resting-state functional magnetic resonance imaging (fMRI) uses coherence in spontaneous activity to map the strength of communication between brain regions (functional connectivity) (15,16). Seminal resting-state work indicates that the brain organizes into intrinsic connectivity networks (ICNs) that support diverse functions (17) and are sensitive to sleep. Lower sleep quality and duration during adolescence are related to weaker connectivity within the default mode network, which supports social cognition, and between the default mode and cognitive control networks (frontoparietal, attention) (18,19). Shorter sleep may also mediate the association between stress and impulsivity, but only for youth with elevated connectivity within the default mode network (20).

While previous studies have focused on the strength of connectivity of specific brain regions, advanced techniques from network neuroscience offer computational techniques to quantify how sleep is associated with parsimonious and interpretable properties of the overall organization of ICNs across the brain (21). Adolescence involves developmental sequences in segregation (communication within ICNs) and integration (communication between ICNs) (22, 23, 24). Segregation yields differentiated networks that execute specialized functions, while integration efficiently coordinates these processing streams across the brain (25). Segregation and integration are reflected in 2 graph theoretical metrics that capture attributes of within-network (within-module degree) and between-network (participation coefficient) connectivity, within the context of the whole-brain system (26). Therefore, graph properties of segregation/integration can provide a parsimonious and developmentally informed account of how sleep is associated with brainwide architecture and behavior.

Several lines of evidence suggest that normative patterns of segregation/integration may be perturbed by shortened sleep in adolescence. Sleep is intertwined with multiple experiences that may calibrate ICN organization, including household instability (27), parenting (28,29), and socioeconomic resources (30, 31, 32). Furthermore, sleep patterns impact developmental processes that partly emerge from segregation and integration, such as psychiatric disorders, multidomain resilience, and executive functioning (33, 34, 35). Lastly, studies have reported preliminary links between sleep patterns and the segregation/integration of ICNs during adolescence (36,37). Nevertheless, no study has integrated a large sample, multimethod sleep assessments, and graph analyses to identify neural signatures of sleep duration in adolescence. Additionally, because sleep is shaped by environmental context (7,38), understanding how other risk factors influence sleep-related neural signatures is critical for prevention and policy. Finally, identifying how these neural signatures impact sleep-related outcomes, such as cognition and psychopathology, can inform intervention.

Therefore, in the current study, we sought to identify graph theoretical signatures of shorter sleep and their relationship with context and behavior. We leveraged the ABCD (Adolescent Brain Cognitive Development) Study, a population-based consortium study of early adolescents with substantial sociodemographic diversity (39). Validation studies indicate that sleep duration may be overestimated by subjective measures and underestimated by objective measures (40,41). Thus, consistent with recommendations and previous studies (42,43), we triangulated objective and subjective sleep assessments (child-report, parent-report, Fitbit) to characterize sleep duration, as well as its association with network segregation/integration and related risk factors and outcomes.

Using multivariate predictive modeling, we found that sleep was robustly related to segregation and integration across the entire brain. Contrary to the current focus on higher-order ICNs (12), we revealed that disconnection of the somatomotor network represents a primary neural signature of shorter sleep. This somatomotor-dominant motif was also linked to key developmental contexts (socioeconomic resources, screen use) and outcomes (psychopathology, cognition). Our study highlights the utility of data-driven, brainwide approaches for elucidating novel neural signatures, and potential intervention targets, of shorter sleep.

## Methods and Materials

### Participants

The ABCD Study is a longitudinal study with 11,875 children (ages 9–10 years) from 22 sites across the United States. The study conforms to procedures of each site’s institutional review board. Participants provide informed consent (parents) or assent (children). This study used ABCD Release 4.0 data, which includes neuroimaging data from baseline (9–10 years) and year 2 (11–12 years). Our primary analysis focused on year 2 data, which has 3 measures of sleep duration. Participants were excluded for failing ABCD quality control (QC), having an insufficient number of resting-state runs each ≥4 minutes after censoring, failing visual QC of registrations and normalizations, and missing data required for analysis (Supplement). This left 5596 participants across 21 sites for our principal component analysis (PCA) identifying components of brain architecture (somatomotor disconnection) (see Results) and 3037 participants with complete data on all sleep measures for examining sleep-brain associations. See the Supplement for sample sizes in brain-context and brain-phenotype analyses (Socioenvironmental Context and Behavioral Phenotypes).

### Functional Neuroimaging

High spatial (2.4 mm isotropic) and temporal resolution (TR = 800 ms) resting-state fMRI was acquired in four 5-minute runs. Preprocessing was performed using fMRIPrep version 1.5.0 (44), including surface reconstruction (FreeSurfer version 6.0.01), spatial normalization, rigid coregistration to the T1-weighted image, motion correction, and transformation to CIFTI space.

We parcellated scans using the Gordon-333 cortical atlas (17), augmented with subcortical (45) and cerebellar (46) atlases. Volumes that exceeded a framewise displacement (FD) threshold of 0.5 mm were censored. After regressing out the linear trend, 24 motion parameters (translations/rotations, derivatives, quadratics), aCompCor 5 cerebrospinal fluid and 5 white matter components and aggressive Independent Component Analysis–based Automatic Removal Of Motion Artifacts (ICA-AROMA) components, high-pass filtering (0.008 Hz), and censoring volumes in a single step, correlation matrices were calculated (see the Supplement).

### Graph Theory

Because most graph measures require unsigned edges, we removed all negative connections, consistent with other investigations (31,32,47,48). We retained all positive connections without additional thresholding given controversies regarding gold-standard thresholding approaches and the cognitive relevance of weak connections (48, 49, 50). We constructed weighted graphs, which resemble biological systems more closely and generate more robust topological measures (48,50,51).

#### Network Segregation

We calculated within-module degree, which captures each node’s connectivity strength within its own network. This metric modifies the module-degree *z* score metric (26) by bypassing within-network *z*-scoring to better capture differences across participants rather than differences across nodes within each network.

#### Network Integration

We calculated the participation coefficient, which captures the diversity of a node’s connections with nodes outside its own network (26). If a node distributes its connectivity evenly across networks, its participation coefficient will be 1, while equality departures yield commensurately lower scores.

We used the community structure defined by the applied parcellation schemes to determine network boundaries. Within-module degree for positive edges (MDP) and participation coefficient for positive edges (PCP) were calculated for 418 nodes, which yielded 836 graph features per participant.

### Sleep Duration

#### Sleep Disturbance Scale

The parent-reported Sleep Disturbance Scale includes 26 questions that assess 6 domains of sleep disturbances over the past 6 months using a 5-point Likert scale (52). This scale has been extensively used in other studies, validly and reliably measures sleep disturbances, and fulfills psychometric requirements for childhood sleep scales. This scale assesses overall sleep duration across weekdays and weekends.

#### ABCD Youth Munich Chronotype Questionnaire

The self-reported ABCD Youth Munich Chronotype Questionnaire includes 17 items that assess diurnal preferences that evoke sleep-wake rhythms (chronotypes), including sleep-wake schedules on school and school-free days (53). This scale has been used in other studies with ABCD data. We generated a weighted average score across weekdays and weekends.

#### Fitbit

Fitbit devices assess biobehavioral features (sleep, physical activity) objectively, continuously, and unobtrusively. Youths wore a Charge 2 Fitbit (Fitbit, Inc.) over 21 days and were instructed to remove it only when charging and bathing (54). The Fitbit app was downloaded on the youth or parent’s phone, and participants were instructed to sync the Fitbit daily and monitor data using *Fitabase*. Sleep intervals were captured by an intrinsic device algorithm. Fitbit measures of sleep have been examined in other studies using ABCD data and have demonstrated adequate sensitivity (55). We generated a weighted average score across weekdays and weekends.

Consistent with established inconsistencies between objective and subjective sleep assessments, these measures exhibited weak-to-moderate agreement (Table S2). We used factor analysis of these 3 measures to construct a latent variable that characterizes each participant’s overall weekly sleep duration across weekdays and weekends (Supplement). This derived sleep factor was very similar to a weighted average of the measures (*r* = 0.99) but accounted for measurement error by capturing shared variance across measures.

### Multivariate Predictive Modeling

To quantify the multivariate relationship between these 836 graph metrics and sleep duration, we conducted principal component regression predictive modeling (56,57) (Supplement). This method applies PCA on the predictive features (MDP/PCP), fits a regression model on the resulting components (determined in nested cross-validation), and implements this model out-of-sample using leave-one-site-out cross-validation (LOSO-CV). We controlled for sex assigned at birth, parent-reported race/ethnicity, age, age^2^, mean FD, and mean FD^2^. Following some recommendations (58), we controlled for race/ethnicity, a social construct, as a proxy for exposure to racism, disadvantage, and opportunity among people of color, variables that were not directly measured (59). The significance of covariate-adjusted models was determined with nonparametric permutation (PERM) tests (60). Exchangeability blocks accounted for twin, family, and site structure using permutation analysis of linear models (61). We visualized results using an importance map and multiplying derived components by Haufe-transformed model betas to promote reliability (62,63), followed by *z* scoring to facilitate interpretation.

### Socioenvironmental Context and Behavioral Phenotypes

Lastly, we examined potential origins and consequences of somatomotor disconnection (Supplement). We investigated key developmental contexts including neighborhood disadvantage (area deprivation index), household income, parental education, and screen time, because socioeconomic conditions and screen time impact sleep duration (64,65). We also assessed key developmental outcomes that are commonly influenced by sleep (5,12), including youth-reported (Brief Problem Monitor) and parent-reported (Child Behavior Checklist) externalizing and internalizing symptoms, general cognitive ability (latent variable from ABCD neurocognitive battery), and grades (School Environment subscale of the School Risk and Protective Factors Survey). We conducted linear regression models predicting each contextual/phenotypic variable from somatomotor disconnection while controlling for sex, race/ethnicity, age, age^2^, mean FD, mean FD^2^, and site.

## Results

### Robust Associations Between Shorter Sleep Duration and Network Segregation/Integration

**Figure 1:**
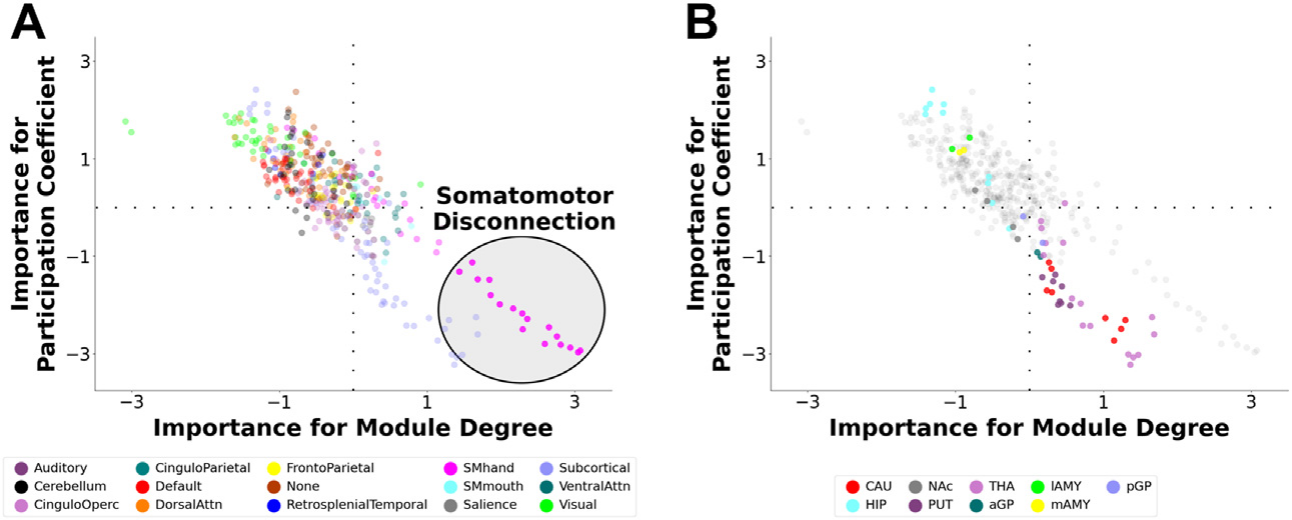
Multivariate neural signature of shorter sleep duration. A multivariate predictive model was trained to predict shorter sleep duration in youths using 836 graph theoretical metrics capturing network segregation (within-module degree) and network integration (participation coefficient) during rest. In leave-one-site-out cross-validation, the model robustly predicted shorter sleep duration in held-out participants. The figure depicts a visualization of the neural signature that was the basis of prediction, in which each brain region was assigned an importance score for within-module degree and participation coefficient based on how important the respective metric was for predicting sleep duration. The sign indicates whether each node’s segregation/integration is positively or negatively related to shorter sleep duration, and the absolute value indicates the importance of the node on that dimension. **(A)** Multivariate neural signature of shorter sleep duration. This map illustrates a prominent motif of somatomotor (SM) disconnection in the lower right, in which among youths with shorter sleep duration, nodes of the SM network exhibit increased within-module degree and reduced participation coefficient. This topological pattern primarily reflects greater segregation (disconnection) of the SM network (with some additional subcortical segregation and visual integration) with shorter sleep duration. **(B)** Multivariate neural signature of shorter sleep duration, focusing on subcortical regions. This map illustrates that the subcortical structures that display greater disconnection with shorter sleep duration primarily involved regions in the caudate nucleus and thalamus. aGP, anterior globus pallidus; Attn, attention; CAU, caudate; HIP, hippocampus; lAMY, lateral amygdala; mAMY, medial amygdala; NAc, nucleus accumbens; Operc, opercular; pGP, posterior globus pallidus; PUT, putamen; THA, thalamus.

We initially predicted shorter sleep duration from 836 indices of network segregation/integration in the year 2 data. The LOSO-CV multivariate relationship was *r*_CV_ = 0.230, *p*_PERM_ < .0001. To characterize the consensus neural signature of shorter sleep, we visualized feature importance (Figure 1). This map demonstrates a prominent role of somatomotor nodes, which exhibited higher segregation and lower integration with shorter sleep duration. Shorter sleep was also associated with lower subcortical integration (particularly thalamus and caudate) and lower segregation of 2 visual nodes. Because the somatomotor network stands out, we parsimoniously describe this motif as somatomotor disconnection.

Next, we implemented the same model to examine the relationship between shorter sleep duration and the full connectome (87,153 connections). The multivariate relationship with the entire connectome (*r*_CV_ = 0.215, *p*_PERM_ < .0001) was slightly lower than with the 836 graph metrics. Thus, we tested whether the graph metrics and the full connectome predicted distinct versus overlapping variance in sleep duration. We built a stacked model by simultaneously entering the predictions from both the graph and connectome models. This model performed similarly to the graph model alone (*r*_cv_ = 0.231), suggesting that despite the 100-fold reduction in the number of features, these graph metrics explain the association between the full connectome and sleep duration.

### A Single Component, Which Exhibits the Somatomotor Disconnection Motif, Is the Dominant Contributor to the Sleep-Brain Relationship

**Figure 2:**
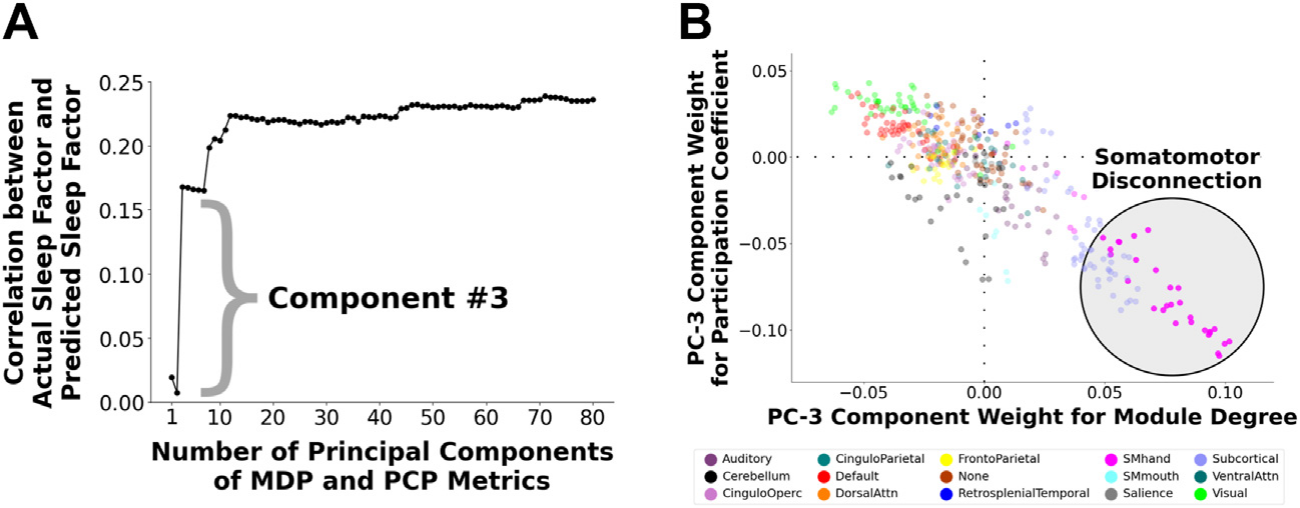
**(A)** Contributions of individual graph theory principal components (PCs) to the multivariate prediction of shorter sleep duration. We conducted a multivariate predictive model to predict shorter sleep duration from 836 nodewise metrics reflecting network segregation (within-module degree for positive connections [MDP]) and network integration (participation coefficient for positive connections [PCP]). We constructed a scree plot to examine contributions of individual components to the multivariate prediction of shorter sleep duration. Component 3 was found to be the dominant contributor in predicting shorter sleep duration. **(B)** Visualization of component 3. We visualized the feature weights for component 3. This component displayed a prominent somatomotor (SM) disconnection motif in which the nodes of the SM network exhibit higher within-module degree and lower participation coefficient (i.e., higher segregation or disconnection than other brain networks). Attn, attention; Operc, opercular.

Our multivariate model applied PCA to 836 graph metrics in year 2, yielding components of interindividual variation predictive of sleep duration. Visualizing each component’s predictivity (Figure 2A) revealed that component 3 was the dominant predictor of sleep duration. This component manifested the somatomotor disconnection motif that summarizes the multivariate signature of shorter sleep (Figure 2B). To a lesser extent, this component also featured greater subcortical segregation, and greater visual and default mode integration, with shorter sleep. The standardized regression coefficient of this somatomotor-centered component with sleep duration was 0.15, which is 67% as strong as the main model using 79 components.

PCA-derived brain components serve as basic units of neural variation that systematically differ across individuals (56). To assess the robustness of our results, we examined how shorter sleep was related to component 3 derived from an independent sample. From the ABCD baseline sample with good resting-state data (*n* = 6570), we repeated our analyses in 3148 youths who did not overlap with our year 2 participants. The baseline component 3 and the year 2 component 3 were nearly identical, exhibiting a prominent somatomotor disconnection motif (Supplement) and highly correlated feature weights (*r* = 0.99).

Second, we calculated the expression of this baseline-derived component 3 for each participant in our year 2 sample by projecting each year-2 participant’s graph metrics onto the coefficients for this component via a vector dot product. The standardized, covariate-adjusted regression coefficient of these scores with sleep duration resembled that from the year 2–derived component (*r* = 0.17), supporting the robustness of this component.

### Associations With Developmental Contexts and Outcomes

Next, we examined how somatomotor disconnection relates to socioenvironmental context and behavior. We focused on the baseline-derived component, ensuring independence between the samples used to identify somatomotor disconnection versus evaluate its nomological network. Because baseline- and year 2–derived components were correlated at *r* = 0.99, results using the year 2–derived component were similar.

**Figure 3:**
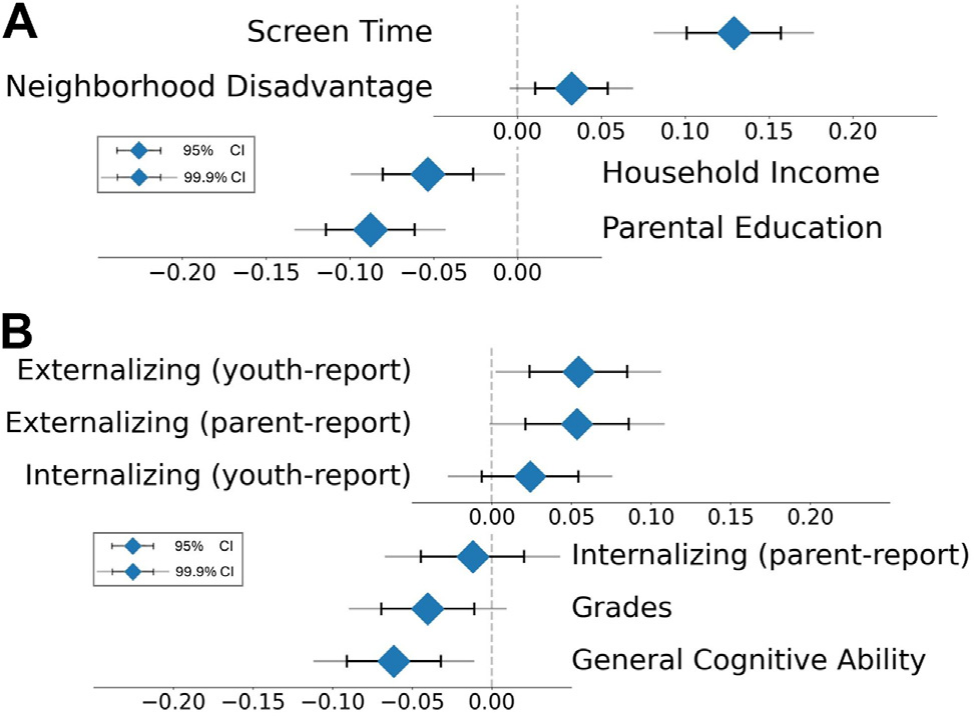
Associations of the sleep-related somatomotor disconnection component with socioenvironmental and behavioral phenotypes. **(A)** Socioenvironmental associations of somatomotor disconnection. Greater disconnection of the somatomotor network was significantly associated with longer screen times, higher neighborhood disadvantage (area deprivation index), lower household income, and lower parental education. **(B)** Phenotypic associations of somatomotor disconnection. Greater disconnection of the somatomotor network was significantly associated with higher youth-reported externalizing problems (Brief Problem Monitor Form), higher parent-reported externalizing problems (Child Behavior Checklist), lower school grades, and lower general cognitive ability (latent variable from ABCD [Adolescent Brain Cognitive Development] Study neurocognitive battery scores). Sleep-related somatomotor disconnection was not significantly associated with youth-reported (Brief Problem Monitor Form) or parent-reported (Child Behavior Checklist) internalizing problems.

As demonstrated in Figure 3, somatomotor disconnection was related to key developmental contexts, including household income (β = −0.05, *p* = 1.0 × 10^−4^), parental education (β = −0.09, *p* = 7.9 × 10^−11^), area deprivation index (β = 0.03, *p* = .003), and screen time (β = 0.13, *p* = 2.6 × 10^−19^). Somatomotor disconnection was also associated with key developmental outcomes, including externalizing symptoms (youth-reported: β = 0.05, *p* = 5.0 × 10^−4^; parent-reported: β = 0.05, *p* = .001), school grades (β = −0.04, *p* = .007), and general cognitive ability (β = −0.06, *p* = 4.8 × 10^−5^) but not internalizing symptoms (youth-reported: β = 0.02, *p* = .119; parent-reported: β = −0.01, *p* = .467).

Next, we tested the association between somatomotor disconnection and shorter sleep when controlling for the socioenvironmental and behavioral variables mentioned above. The relationship remained significant (β = −0.13, *p* = 2.1 × 10^−9^), indicating that somatomotor disconnection was uniquely related to sleep duration, even after accounting for intertwined exposures and phenotypes.

### Sensitivity Analyses

**Figure 4:**
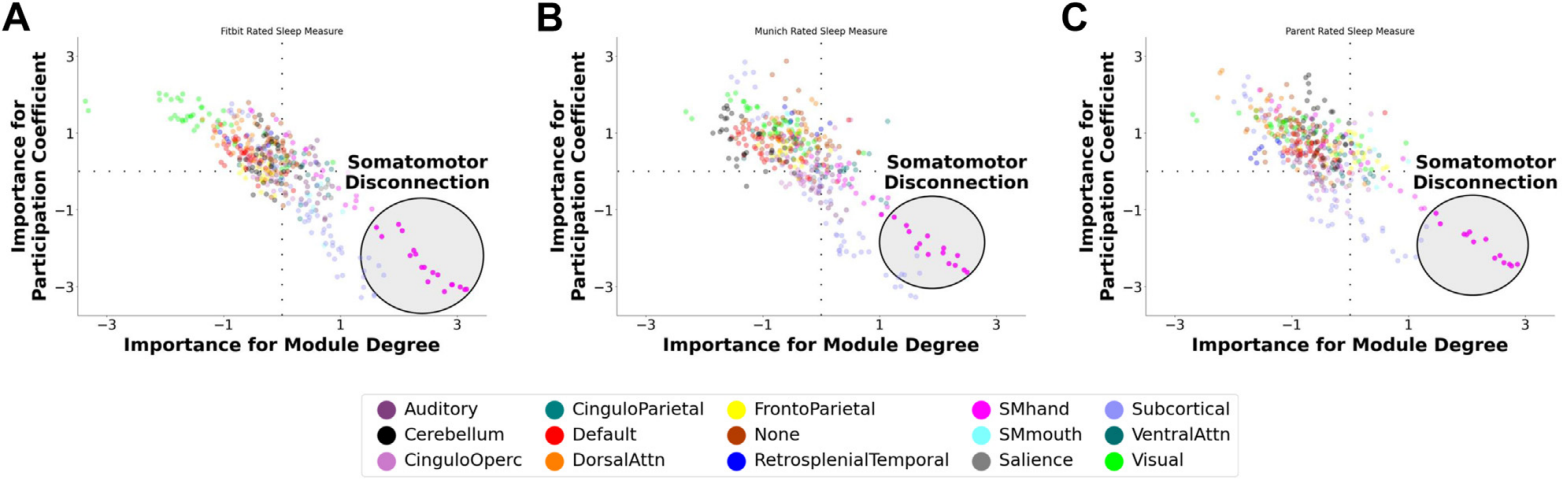
Multivariate neural signature of shorter sleep duration broken down by method of sleep measurement. The figure depicts a visualization of the neural signature that predicted shorter sleep duration, as measured by **(A)** Fitbit wearables, **(B)** youth reports, and **(C)** parent reports. Each brain region was assigned an importance score for within-module degree and participation coefficient based on how important the respective metric was for predicting sleep duration. The sign indicates whether each node’s segregation/integration was positively or negatively related to shorter sleep duration, and the absolute value indicates the importance of the node on that dimension. Regardless of the method of sleep duration measurement, this map illustrates a prominent motif of somatomotor (SM) disconnection in the lower right, in which among youths with shorter sleep duration, nodes of the SM network exhibit increased within-module degree and reduced participation coefficient. This topological pattern primarily reflects greater segregation (disconnection) of the SM network (with some additional subcortical segregation and visual integration) with shorter sleep duration. Attn, attention; Operc, opercular.

Finally, we confirmed the robustness of our results with 2 sensitivity analyses (Supplement). First, we repeated our analyses with each indicator of sleep duration separately (youth-report, parent-report, Fitbit). Multivariate associations with brain architecture were strongest for the latent variable, followed by Fitbit. Nevertheless, the consensus neural signature for each sleep indicator (following Haufe transformation) consistently exhibited the characteristic somatomotor disconnection motif (Figure 4). Second, given controversies regarding the use of race/ethnicity as a proxy for experiences (58,66), we refit our models without controlling for race/ethnicity. Results were remarkably consistent, indicating that our findings are robust to methodological variation.

## Discussion

Shorter sleep increases risk for impaired regulation over behavior, cognition, and emotion (1,3,5); is especially common in adolescence (6); disrupts brain development (12); and incurs substantial societal costs (67). No study has identified parsimonious brain signatures underlying shortened sleep and its neurobehavioral sequelae during this critical developmental period. By integrating techniques from network science and multivariate modeling in a large, sociodemographically diverse cohort, we elucidated a novel neural signature of shorter sleep in adolescence that mainly reflects greater disconnection of the somatomotor network. This sleep-related architectural motif was also associated with socioeconomic context, screen time, cognition, and psychopathology. This brain signature could serve as a novel marker for early risk identification, extends our understanding of how environmental insults confer risk, and may inform new psychosocial and neuromodulatory interventions.

### Multivariate Link Between Sleep Duration and Network Segregation/Integration

Given established disagreements between subjective and objective measures of sleep (68), we triangulated subjective (self- and parent-report) and objective (Fitbit) assessments to construct a multimodal index of sleep duration. Similar to previous work (69), the out-of-sample correlation between sleep duration and the full connectome was 0.22. While this effect is relatively large for context-brain associations, multivariate techniques describing how sleep duration relates to 87,153 connections do not afford clear interpretations of what these neural alterations mean and how they are organized across the brain.

Here, we leveraged graph theory to distill these 87,153 connections into only 836 features with neuroscientific interpretability. We quantified each region’s integration (participation coefficient) and segregation (within-module degree). Despite this 100-fold reduction in the number of features, we still captured the entire association between sleep duration and brain organization. These findings suggest that network segregation/integration offer a parsimonious and interpretable framework for understanding how adolescent sleep duration relates to neural architecture.

### Somatomotor Disconnection as a Neural Signature of Shorter Sleep

Current perspectives predominantly focus on how sleep impacts higher-order brain systems. These systems include the default mode network (18,19,36), its interactions with cognitive control networks (frontoparietal, attention) (19), and corticolimbic circuitry implicated in emotional learning and regulation (43). Against this backdrop, we expected the brainwide signature of shorter sleep to primarily feature association systems. However, the defining characteristics of this signature involved high segregation and low integration of a lower-order system, i.e., the somatomotor network, spanning precentral/postcentral gyri extending into supplementary motor cortex. Shorter sleep was also associated with lower integration of subcortical regions that underlie motor control, sensory integration, and reward learning (thalamus, caudate) (70,71), as well as lower segregation of 2 visual regions. This gradient of greater somatomotor segregation but greater visual integration suggests a spatial patterning of sleep-brain associations along the visual-to-motor axis, the primary axis of macroscale organization at this age (72). However, because the somatomotor network stands out in this multivariate signature, we parsimoniously describe this motif as somatomotor disconnection. That is, with shorter sleep, regions in the somatomotor network became 1) more interconnected and 2) less connected with other systems, making them more disconnected in the whole-brain architecture.

This somatomotor-dominant motif was robust to measurement variation, because it also characterized the neural signature for each individual sleep indicator (youth-report, parent-report, Fitbit). Furthermore, of 79 components of brain architecture, 1 component was the dominant contributor to the multivariate relationship between sleep duration and brainwide organization, which again reflected somatomotor disconnection. Finally, we replicated this result using an independent sample and while controlling for related contexts (socioeconomic resources, screen time) and phenotypes (cognition, psychopathology), thereby confirming the internal structure, developmental stability, and generalizability of somatomotor disconnection.

Converging evidence from innovative techniques such as graph theory (30,31,34), polyneuro risk profiles (73), and multivariate modeling (57,74) demonstrates that context and phenotypes are associated with the organization of the entire brain. Therefore, it is highly uncommon, and thus particularly remarkable, that the topology of a single network captures the bulk of variance in sleep duration. Notably, this network has not been commonly considered in the literature, which instead has reported dominant associations between sleep and default mode network connectivity; however, these studies have only investigated the default mode network (18,20) or have had small samples (75). Accordingly, while theoretically driven approaches delineate the role of specific brain systems, our findings illustrate that data-driven brainwide techniques in large-scale studies can uncover the central role of previously understudied systems.

The central involvement of the somatomotor network in sleep may initially seem surprising. However, inhibitory neurotransmission in the substantia nigra, a subcortical structure critical for motor control, regulates sleep in rodents (76). Sleep quality has also been associated with intrinsic connectivity of precentral and supplementary motor areas (69,77). Lastly, sleep quality and duration have been linked to somatomotor network integration (37), supporting the critical involvement of the somatomotor system in sleep.

We offer several speculations regarding the neurophysiological implications of somatomotor disconnection. First, key motor areas such as the basal ganglia regulate sleep via neurochemical and electrophysiological mechanisms (78). Thus, somatomotor dysconnectivity may indicate impaired regulation over sleep activity. Second, sleep and wakeful states differ in levels of motor activity, which may become expressed along the somatomotor network. Third, sleep may be most strongly related to systems with the highest plasticity during each developmental period, which include somatomotor systems earlier in adolescence but association systems later on (79,80). Additional research is required to parse whether somatomotor disconnection constitutes a cause, marker, risk factor, or consequence of shorter sleep during adolescence.

### Contextual and Phenotypic Implications of Somatomotor Disconnection

We also characterized how sleep-related somatomotor disconnection relates to critical developmental contexts and outcomes. We found stronger somatomotor disconnection in youths who spent more time on their screens. One interpretation is that longer screen times take time away from sleep (12), magnifying sleep-related neural signatures. Moreover, we observed stronger somatomotor disconnection in youths with higher neighborhood disadvantage, lower household income, and lower parental education. This finding is consistent with evidence of poorer sleep in disadvantaged communities, partially due to higher average exposure to stress or ambient noise (65), and with consistent links among somatomotor connectivity and multiple forms of stress exposure (30,32,81). For example, we recently demonstrated that lower socioeconomic resources are associated with greater somatomotor segregation in the ABCD Study (32). These findings suggest that somatomotor disconnection may represent a robust neural marker of diverse forms of environmental risk and opportunity.

Greater sleep-related somatomotor disconnection was also linked to lower general cognition and school grades. While cognition is generally attributed to higher-order association systems, cognitive functions are emergent properties of activity across the entire brain, including lower-order systems (22,82). Across development, the somatomotor network becomes more integrated with other systems to coordinate specialized neural computations required for complex cognition (23,24). The somatomotor network also interacts with regulatory networks to translate abstract cognitive representations into goal-relevant behavior (83). Finally, somatomotor dysconnectivity relates to cognitive dysfunction in adults (84). Thus, sleep-related somatomotor disconnection may undermine cognitive performance in experimental and naturalistic settings.

Lastly, dovetailing with evidence that disrupted sleep heightens psychiatric vulnerability (1,3,4), we found that greater somatomotor disconnection was associated with elevated externalizing symptoms. Individual and meta-analytic investigations have linked somatomotor function to psychiatric symptoms across the lifespan (85,86). Somatomotor dysconnectivity may reflect motor impulsiveness that confers shared liability for various forms of externalizing behavior (85). Motor systems have also been implicated in several transdiagnostic processes, including emotion regulation (87) and impulsivity (84). Additional research is required to uncover the mechanisms through which somatomotor disconnection shapes cognition and psychopathology.

### Limitations and Future Directions

Limitations and recommendations for future research are important to consider. First, we focused on sleep duration given its clear implications for policy and prevention. However, other aspects of sleep (e.g., quality, consistency) may implicate different brain systems and are important to examine in future studies. Second, our main analyses were cross-sectional during the wave with 3 sleep duration measures, restricting inferences about direction of causality or patterns of neurodevelopment. Longitudinal investigations across longer time scales should be used to evaluate whether somatomotor disconnection precedes or follows shorter sleep and how it unfolds across time. Third, the extent to which our results generalize across community populations with different sociodemographic backgrounds and clinical populations with more severe sleep disruptions remains unclear (88,89). Fourth, future studies should confirm somatomotor disconnection using alternative measures of network segregation/integration (e.g., connectivity strength). Lastly, relationships between somatomotor disconnection and phenotypic outcomes were relatively modest in the current study, highlighting the importance of other topological signatures.

### Conclusions

In the current study, we uncovered a novel, generalizable, and robust neural signature of shortened sleep during adolescence. Specifically, shorter sleep was related to greater expression of a topological motif primarily defined by disconnection of the somatomotor network, which in turn was linked to lower socioeconomic resources, longer screen times, reduced cognitive performance, and elevated externalizing problems. These neural markers of shorter sleep may inform early risk identification and targeted interventions to scaffold healthy cognitive, emotional, and brain development across adolescence.

## Supplementary Material

Supplemental Methods and Results, Figures S1–S5, Tables S1–S2, and fMRIPrep Details

Key Resources Table
