# Supplementary material for: Somatomotor Disconnection Links Sleep Duration With Socioeconomic Context, Screen Time, Cognition, and Psychopathology
Source: Biol Psychiatry Glob Open Sci. 2025 Apr 30;5(4):100522. doi: 10.1016/j.bpsgos.2025.100522 (PMC12172974; doi:10.1016/j.bpsgos.2025.100522)
Supplement: Supplemental Methods and Results, Figures S1–S5, Tables S1–S2, and fMRIPrep Details [file mmc1.pdf]

SUPPLEMENTARY INFORMATION

Somatomotor Disconnection Links Sleep Duration With Socioeconomic Context, Screen Time, Cognition, and Psychopathology

Michael *et al.*

Supplemental Methods and Results

1. Sample and Data

The ABCD study is a multisite longitudinal study with 11,875 children between 9-10 years of age from 22 sites across the United States. The study conforms to the rules and procedures of each site's Institutional Review Board, and all participants provide informed consent (parents) or assent (children). Data for this study are from ABCD Release 4.0.

2. Data Acquisition, fMRI Preprocessing, and Connectome Generation

Imaging protocols were harmonized across sites and scanners. High spatial (2.4mm isotropic) and temporal resolution (TR = 800ms) resting-state fMRI was acquired in four separate runs (5min per run, 20min total). The entire data pipeline described below was run through automated scripts on the University of Michigan's high-performance cluster.

Preprocessing was performed using fMRIPrep version 1.5.0 (1), and detailed methods automatically generated by fMRIPrep software are provided in the fMRIPrep Supplement below. T1-weighted (T1w) and T2-weighted images were run through recon-all using FreeSurfer v6.0.1. T1w images were also spatially normalized nonlinearly to MNI152NLin6Asym space using ANTs 2.2.0. Each functional run was corrected for fieldmap distortions, rigidly coregistered to the T1, motion corrected, and normalized to standard space. ICA-AROMA was run to generate aggressive noise regressors. Anatomical CompCor was run and the top 5 principal components of both CSF and white matter were retained. Functional data were transformed to CIFTI space using HCP's Connectome Workbench. All preprocessed data were visually inspected at two separate stages to ensure only high-quality data was included: after co-registration of the functional data to the structural data and after registration of the functional data to MNI template space.

Connectomes were generated for each functional run using the Gordon 333 parcel atlas (2), augmented with parcels from high-resolution subcortical (3) and cerebellar (4) atlases. Volumes exceeding a framewise displacement threshold of 0.5mm were marked to be censored. Covariates were regressed out of the time series in a single step, including: linear trend, 24 motion parameters (original translations/rotations + derivatives + quadratics), aCompCorr 5 CSF and 5 WM components and ICA-AROMA aggressive components, high-pass filtering at 0.008Hz, and censored volumes. Next, correlation matrices were calculated for each run. Each matrix was then Fisher r-to-z transformed, and then averaged across runs for each participant to yield their final connectome.

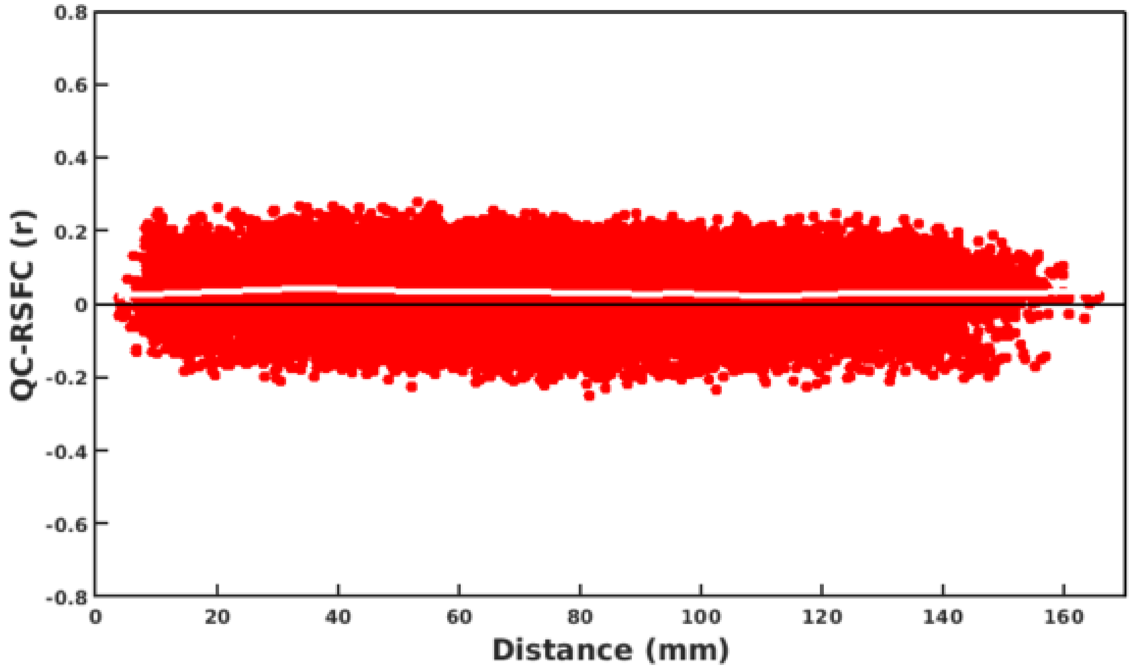

Figure S1. Quality Control Resting-State Functional Connectivity Plot

We used multiple procedures listed above to limit the effect of head motion on resting-state functional connectivity maps. To assess the effectiveness of these procedures, we produced a quality control resting-state functional connectivity (QC-RSFC) plot (5,6). This plot shows the relationship between mean framewise displacement and connectivity edges binned by distance. Motion effects produce a sloped line (distance-dependent artifact), while a flat line is indicative of minimal motion-related effects. The QC-RSFC plot for our ABCD resting-state data showed a flat line (Figure S1), providing additional evidence that our stringent motion correction strategies were effective.

3. Inclusion/Exclusion

There are 11,875 participants in the ABCD Release 4.0 dataset. Screening was initially done using ABCD raw QC to limit to participants with 2 or more good runs of resting data as well as a good T1 and T2 image (QC score, protocol compliance score, and complete all = 1). Each run was visually inspected for registration and warping quality, and only those participants who still had 2 or more good runs were retained. After connectome generation, runs were excluded if they had less than 4 minutes of uncensored data, and next participants were retained only if they had 2 or more good runs (N = 5,596). Finally, participants who were missing data required for factor modeling of sleep were dropped from that analysis, leaving N = 3,037. Demographic characteristics of the excluded and included sample are shown in Table S1.

	Included Sample
N	3037
Age (mean (s.d.))	11.95 (0.65)
Female (%)	1526 (50.2)
Race-Ethnicity (%)	
non-Hispanic White	1832 (60.3)
non-Hispanic Black	251 (8.3)
Hispanic	597 (19.7)
non-Hispanic Asian	57 (1.9)
Multi-racial/Other	300 (9.9)
No answer	–
Average Parental Education (%)	
< HS Diploma	78 (2.6)
HS Diploma/GED	195 (6.4)
Some College	717 (23.6)
Bachelor	858 (28.3)
Post Graduate Degree	1185 (39.0)
No answer	4 (0.1)
Household Marital Status – Married (%)	2222 (73.2)
Household Income (%)	
<50K	608 (20.0)
>=50k & <100K	800 (26.3)
>=100k	1520 (50.0)
No answer	109 (3.6)

Table S1. Demographic Characteristics of Included Participants

4. Graph Theoretic Measures of Functional Brain Architecture

For each participant, we calculated network segregation (within-module degree) and network integration (participation coefficient) from their weighted functional connectivity matrices, focusing on positive connections.

Within-module degree is a graph theoretic measure of within-network connectivity. Formally, the within-module degree of a node i is given by:

$$\sum_{j=1}^{N_i} e_{ij}$$

where e_{ij} is the edge weight between nodes i and j, and N_i is the set of nodes incident to node i that are in the same network as i.

Participation coefficient is a graph theoretic measure of between-network connectivity. Formally, the participation coefficient of a node i is given by:

$$1 - \sum_{m}^{M} \left(\frac{e_i(m)}{e_i}\right)^2$$

where M is the set of networks, $e_i(m)$ is the sum of edge weights between node i and all nodes in network m and e_i is the sum of edge weights between node i and all other nodes.

5. Principal Components Regression-Based Multivariate Predictive Modeling

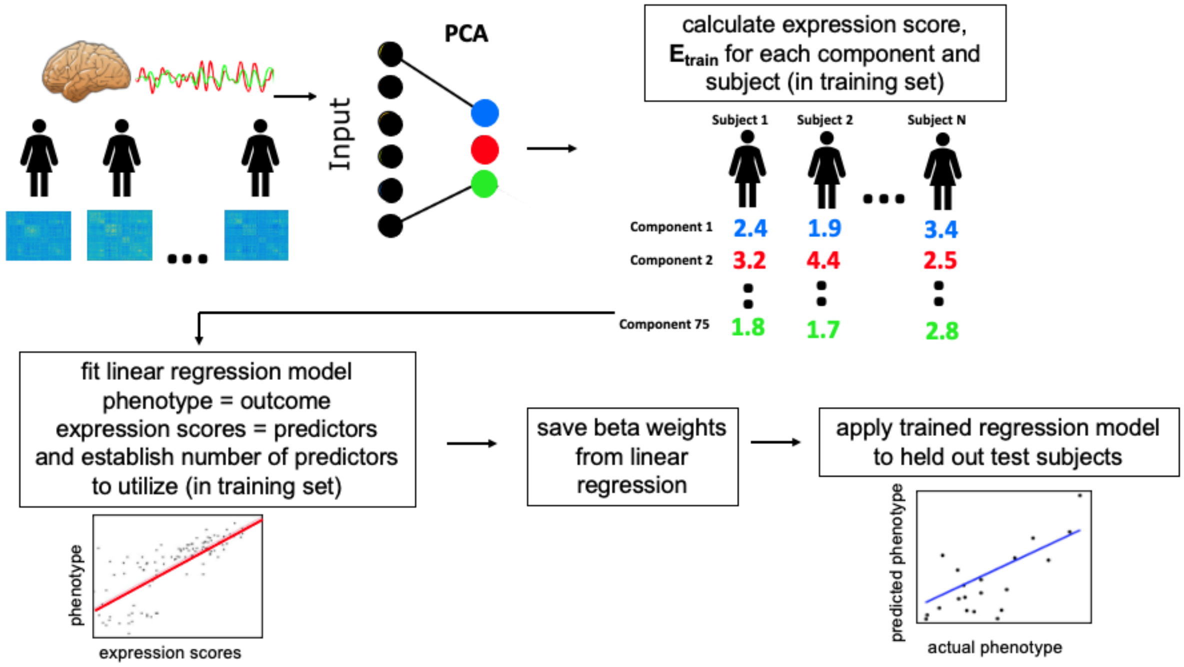

Figure S2. Steps of Principal Component Regression Predictive Modeling.

We implemented principal component regression (PCR) (7) as a multivariate predictive modeling method for identifying brain-behavior relationships (8) (see Figure S2). The method involves two key steps: 1) Use principal component analysis (PCA) to find a set of components that capture *inter-individual* differences in brain features; 2) Use multiple regression in a cross-validation framework to link expression scores for these components to phenotypes of interest. In previous work, we often used the more general name brain basis set (BBS) for this approach to capture commonalities with work by our group and others that use alternative methods for step 1 (e.g., independent component analysis (9,10) or community detection (11,12)). We chose the PCR approach for this study because our previous work showed it has high test-retest reliability (13) and predictive accuracy (14,15) and generally performs as well as or better than alternative methods such as support vector regression and ridge regression (13).

We performed PCA dimensionality reduction on an *n* subjects by *p* connectivity features matrix, yielding *n* principal components (i.e., directions in the feature space) that represent inter-individual differences in the imaging features (functional connectivity or graph theory metrics). Pre-subject expression scores for a subset of *k* of these components then entered multiple regression modeling to identify linear associations with phenotypes of interest (here, sleep duration). Of note, we selected *k* using 5-fold cross-validation within the training data, as in our previous work (13).

To assess accuracy and generalizability of PCR predictive models, we used leave-one-site-out cross-validation. In each fold of the cross-validation, data from one of the 21 sites served as the held-out test dataset and data from the other 20 sites served as the training dataset. Additionally, to ensure separation of train and test datasets, at each fold of the cross-validation, a new PCA was performed on the imaging features (functional connectivity or graph theory metrics) in the training dataset, and expression scores of these brain components were calculated for the test set. Note that by employing leave-one-site-out, members of twinships and sibships are never present in both training and test samples. We assessed the performance of PCR predictive models with cross-validated Pearson's correlation and cross-validated partial eta squared.

In each fold of the leave-one-site out cross-validation (LOSO-CV), PCR predictive models were trained in the train partition with the following covariates (unless explicitly stated otherwise for specific analyses): sex, race-ethnicity, age, age squared, mean FD and mean FD squared. To maintain strict separation between training and test datasets, regression coefficients for the covariates learned from the training sample were applied to the test sample to calculate effect size measures (Pearson's $\text{correlation}_{\text{cross-validated}}$). This procedure is described in detail in our previous publications (15,16).

We assessed the significance of all cross-validation-based correlations with non-parametric permutation tests. We randomly permuted the 5,596 participants' outcome variable values 10,000 times and reran the PCR predictive modeling stream at each iteration, yielding a null distribution of correlation values. The procedure of Freedman and Lane (17) was used to account for covariates. In addition, exchangeability blocks were used to account for twin, family, and site structure and were entered into Permutation Analysis of Linear Models (PALM) (18) to produce permutation orderings.

6. Latent Variable Modeling for Sleep Duration

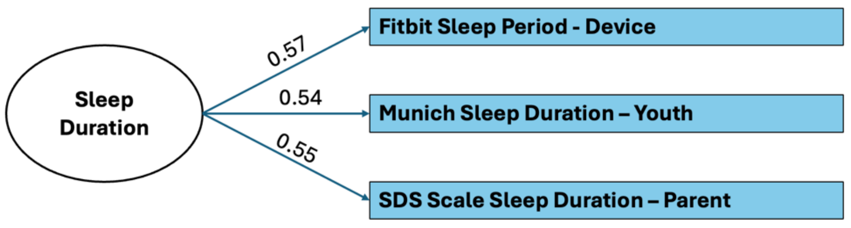

Figure S3. Factor Model of Sleep Duration. *Path estimates reflect standardized factor loadings.*

To construct a latent variable for sleep duration that triangulates across objective (Fitbit) and subjective (parent- and youth-report) assessments, we conducted a factor analysis using the "fa" function from the psych package (version 2.4.6.26) in R (version 4.3.2). Since the model only has three indicators, it is just-identified; therefore, model fit indices are not available.

For the parent-reported Sleep Disturbance Scale (SDSC), we used the sleep duration item. This item asks "How many hours of sleep does your child get on most nights? 1 = 9-11 hours; 2 = 8-9 hours; 3 = 7-8 hours; 4 = 5-7 hours; 5 = Less than 5 hours. For each response, we assigned the midpoint of the associated range, and assigned 5 for the "Less than 5 hours response". For the youth-rated Munich Chronotype Questionnaire, we determined sleep duration as the difference between (a) mctq_sow_calc (Sleep onset, workday) and mctq_sd_wake_up_time_calc (Sleep end, workday) and (b) mctq_sof_calc (Sleep onset, freeday) and mctq_fd_wake_up_time_calc (Sleep end, freeday), and calculated a weighted average. For the Fitbit data, we used the fit_ss_sleepperiod_minutes variable during both weekends and weekdays, and calculated a weighted average.

**Sleep Variable**	fitbit_weekdays	fitbit_weekends	mctq_weekdays	mctq_weekends
fitbit_weekdays	-	-	-	-
fitbit_weekends	.45	-	-	-
mctq_weekdays	.27	.17	-	-
mctq_weekends	.16	.15	.30	-
sdsc_overall	.31	.20	.29	.13

Table S2. Zero-Order Correlations among Sleep Duration Variables. *fitbit_weekdays and fitbit_weekends represent sleep duration during weekdays or weekends, respectively, as assessed using Fitbit measures. mctq_weekdays and mctq_weekends represent sleep duration during weekdays or weekends, respectively, as assessed using the youth-reported Munich Chronotype Questionnaire. sdsc_overall represents sleep duration across weekdays and weekends, as assessed using the parent-reported Sleep Disturbance Scale.*

7. Additional Socio-Environmental and Behavioral Variables

We examined associations between expression of a somatomotor disconnection graph theory component and a number of socio-environmental variables from the ABCD dataset. The number of participants included in this analysis varied depending on the availability of the variable. We provide additional information on these variables and the sample size for the analysis below.

Household Income (n=5,342) - This variable covered all sources of income for family members, including wages, benefits, child support payments, and others. It was assessed in bins as follows: 1 = <5,000, 2 = 5,000 - 11,999, 3 = 12,000 - 15,999, 4 = 16,000 - 24,999, 5 = 25,000 - 34,999, 6 = 35,000 - 49,999, 7 = 50,000 - 74,999, 8 = 75,000 - 99,999, 9 = 100,000 - 199,999, 10 = More than 200,000. As this variable involves ordinal representations of a continuous measure, we assigned each participant the midpoint for their bin to enter household income as a continuous variable in regression analyses, consistent with previous work in the ABCD Study (19).

Parental Education (*n*=5,582) - This variable reflects the average educational achievement of parents or caregivers, and it is recoded into years based on the method of a previous ABCD report (19).

Area deprivation index (n=5,262) - This variable reflects an ABCD consortium-supplied variable (reshist_addr1_adi_wsum). Higher scores on the index indicate greater deprivation including higher percent of families living in poverty, increased unemployment, and lower levels of educational attainment at the neighborhood level.

Child's screen time (*n*=5,590) - Youth participants estimated the amount of time they spent on each of a series of six digital activities on both "typical" weekday and weekend days, which were then weighted (5*weekday + 2*weekend) to yield a total screen estimate.

Parent-reported externalizing and internalizing symptoms (*n*=4,704) - Parents completed the *Child Behavior Checklist (CBCL)*. The CBCL includes two overarching scales. The Internalizing Problems composite scale is composed of the Withdrawn, Somatic Complaints, and Anxious/Depressed subscales. The Externalizing Problems composite scale is composed of the Delinquent Behavior and Aggressive Behavior subscales.

Youth-reported externalizing and internalizing symptoms (*n*=5,394) - Youth completed the *Brief Problem Monitoring Form*, an abbreviated 18-item version of the CBCL, which has internalizing and externalizing composite scores.

Child's general cognitive ability (n=4,445) - We created a general cognitive ability variable from bifactor modeling of the ABCD neurocognitive battery. Details on the creation of the variable and predictive validation of the variable are available in our previous studies in ABCD (16,20).

Child's grades (n=5,377) - Parents completed the *School Risk and Protective Factors Survey*, and child's grades were drawn from the *School Environment* subscale. We investigated youth-reported (sag_grades_last_yr) and parent-reported (sag_grade_type) overall grades over the last year. As this variable involves ordinal representations of a continuous measure, for each reporter,

each response was assigned the midpoint of the associated range (e.g., a score of 1, indicating 97-100, was assigned the score of 98.5) to enter school grades as a continuous variable in regression analyses. We then calculated the average of the youth-reported and parent-reported overall grades as the final score.

8. Comparison of the Somatomotor Disconnection Component Across Two Independent ABCD Samples

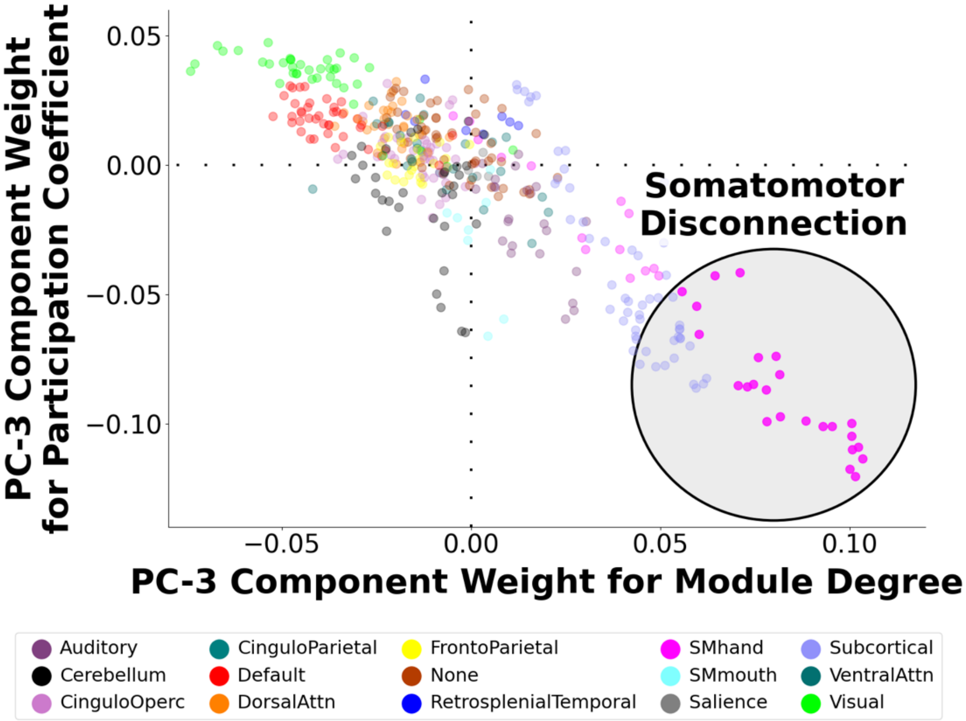

Figure S4. Visualization of component #3 derived from the ABCD baseline sample. *The component, which exhibits a prominent somatomotor disconnection motif, is highly similar to component #3 from the ABCD year-2 sample.*

We obtained component #3 from graph theoretic metrics from 3,148 participants from the ABCD baseline sample who have no overlap with the wave 2 participants used in the main analysis. We found that component #3 from this distinct sample was nearly identical to component #3 from the year-2 sample (see Figure S4), even though there was no overlap in the participants in the two samples. Indeed, feature weights for baseline- and year 2-derived components displayed a strong correlation of 0.99.

9. Analyses with Individual Sleep Duration Variables

We next repeated our analyses with each indicator of our sleep duration factor (objective Fitbit, subjective youth-report, subjective parent-report) to identify whether different brain signatures

are implicated in perceived versus objective measures of sleep duration (*N* = 3,037). The LOSO-CV multivariate relation varied across Fitbit measures (MDP/PCP model = 0.176, connectome model = 0.207, stacked model = 0.205), youth-reported measures (MDP/PCP model = 0.122, connectome model = 0.099, stacked model = 0.118), and parent-reported measures (MDP/PCP model = 0.119, connectome model = 0.122, stacked model = 0.130). However, as expected, these findings demonstrate stronger multivariate associations with our latent measure of sleep duration.

10. Analyses without Race/Ethnicity as a Covariate

Finally, we repeated our analyses after excluding race/ethnicity as a covariate from our models. These analyses revealed even stronger associations between sleep duration and functional brain architecture. Specifically, the LOSO-CV multivariate relationship was r_{cv}=0.294 for the MDP/PCP model, r_{cv}=0.309 for the connectome model, and r_{cv}=0.317 for the stacked MDP/PCP and connectome model. The Haufe-transformed consensus neural signature from the multivariate model again revealed a prominent pattern of greater somatomotor disconnection as a function of shorter sleep duration (**Figure S5A**). Visualizing each component's predictivity (**Figure S5B**) again indicated that the somatomotor disconnection-centered motif (component #3) was the dominant contributor in predicting sleep duration when ethnoracial identity was not included in the models. Finally, greater somatomotor disconnection was again associated with important developmental contexts (longer screen times, lower household income, lower parent education, higher neighborhood disadvantage) and developmental outcomes (lower school grades, lower general cognitive ability, elevated externalizing symptoms), but was unrelated to internalizing symptoms (**Figure S5C**), closely mirroring our primary analysis.

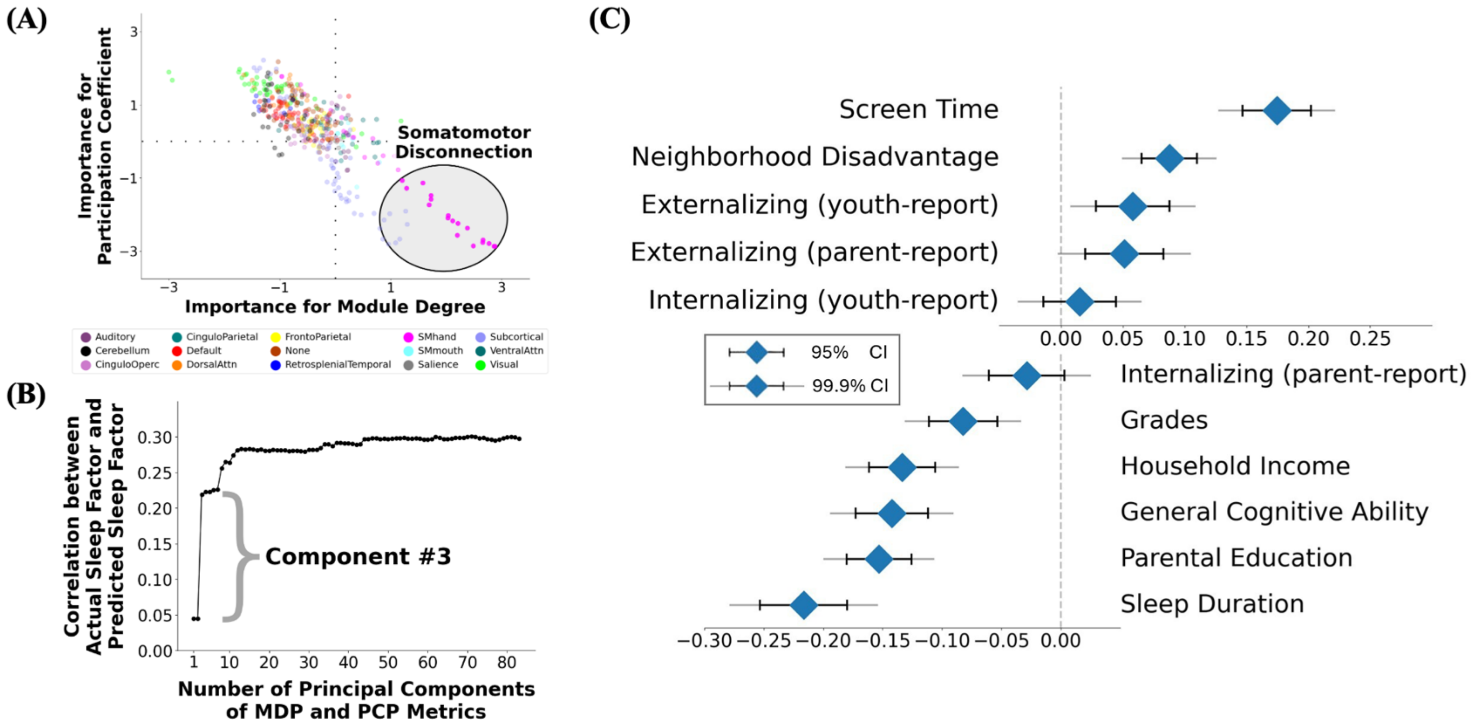

Figure S5. Sensitivity Analyses without Race/Ethnicity as a Covariate. (A) Multivariate neural signature of shorter sleep duration. *This map illustrates a prominent motif of somatomotor disconnection in the lower right, in which among youth with shorter sleep duration, nodes of the somatomotor network exhibit increased within-module degree and reduced participation coefficient. This topological pattern primarily reflects greater segregation ("disconnection") of the somatomotor network with shorter sleep duration.* ***(B) Contributions of individual graph theory principal components (PC) to the multivariate prediction of shorter sleep duration****. The scree plot demonstrates that Component #3, which predominantly reflects the somatomotor disconnection-centered motif, was the dominant contributor in predicting shorter sleep duration.* ***(C) Associations of the sleep-related somatomotor disconnection component with socio-environmental and behavioral phenotypes****. Greater somatomotor disconnection was associated with longer screen times, lower socioeconomic resources, lower cognitive ability, lower school grades, and elevated externalizing problems.*

References

1. Esteban O, Markiewicz CJ, Blair RW, Moodie CA, Isik AI, Erramuzpe A, *et al.* (2019): fMRIPrep: A robust preprocessing pipeline for functional MRI. *Nat Methods* 16: 111–116.
2. Gordon EM, Laumann TO, Adeyemo B, Huckins JF, Kelley WM, Petersen SE (2016): Generation and evaluation of a cortical area parcellation from resting-state correlations. *Cereb Cortex* 26: 288–303.
3. Tian Y, Margulies DS, Breakspear M, Zalesky A (2020): Topographic organization of the human subcortex unveiled with functional connectivity gradients. *Nat Neurosci* 23: 1421–1432.
4. Diedrichsen J, Maderwald S, Küper M, Thürling M, Rabe K, Gizewski ER, *et al.* (2011): Imaging the deep cerebellar nuclei: A probabilistic atlas and normalization procedure. *NeuroImage* 54: 1786–1794.
5. Power JD, Mitra A, Laumann TO, Snyder AZ, Schlaggar BL, Petersen SE (2014): Methods to detect, characterize, and remove motion artifact in resting state fMRI. *NeuroImage* 84: 320–341.
6. Power JD, Schlaggar BL, Petersen SE (2015): Recent progress and outstanding issues in motion correction in resting state fMRI. *NeuroImage* 105: 536–551.
7. Jolliffe IT (1982): A note on the use of principal components in regression. *J R Stat Soc Ser C Appl Stat* 31: 300–303.
8. Sripada C, Angstadt M, Rutherford S, Kessler D, Kim Y, Yee M, Levina E (2019): Basic units of inter-individual variation in resting state connectomes. *Sci Rep* 9: 1900.

9. Kessler D, Angstadt M, Sripada C (2016): Growth charting of brain connectivity networks and the identification of attention impairment in youth. *JAMA Psychiatry* 73: 481–489.

10. Kessler D, Angstadt M, Welsh RC, Sripada C (2014): Modality-spanning deficits in attention-deficit/hyperactivity disorder in functional networks, gray matter, and white matter. *J Neurosci Off J Soc Neurosci* 34: 16555–16566.

11. Arroyo J, Athreya A, Cape J, Chen G, Priebe CE, Vogelstein JT (2021): Inference for multiple heterogeneous networks with a common invariant subspace. *J Mach Learn Res* 22: 1–49.

12. Levin K, Lodhia A, Levina E (2021): Recovering shared structure from multiple networks with unknown edge distributions. *arXiv.org*. Retrieved August 30, 2024, from https://www.proquest.com/docview/2243263148?parentSessionId=DY7JPHVT%2FEv8cCXZsoUmKb1fytpZEe1ztfHFS0n%2B6fo%3D&pq-origsite=primo&sourcetype=Working%20Papers

13. Taxali A, Angstadt M, Rutherford S, Sripada C (2021): Boost in test–retest reliability in resting state fMRI with predictive modeling. *Cereb Cortex* 31: 2822–2833.

14. Sripada C, Rutherford S, Angstadt M, Thompson WK, Luciana M, Weigard A, *et al.* (2020): Prediction of neurocognition in youth from resting state fMRI. *Mol Psychiatry* 25: 3413–3421.

15. Sripada C, Angstadt M, Rutherford S, Taxali A, Shedden K (2020): Toward a “treadmill test” for cognition: Improved prediction of general cognitive ability from the task activated brain. *Hum Brain Mapp* 41: 3186–3197.

16. Sripada C, Angstadt M, Taxali A, Clark DA, Greathouse T, Rutherford S, *et al.* (2021): Brain-wide functional connectivity patterns support general cognitive ability and mediate effects of socioeconomic status in youth. *Transl Psychiatry* 11: 1–8.

17. Freedman D, Lane D (1983): A nonstochastic interpretation of reported significance levels. *J Bus Econ Stat* 1: 292–298.

18. Winkler AM, Renaud O, Smith SM, Nichols TE (2020): Permutation inference for canonical correlation analysis. *NeuroImage* 220: 117065.

19. Rakesh D, Zalesky A, Whittle S (2021): Similar but distinct – effects of different socioeconomic indicators on resting state functional connectivity: Findings from the Adolescent Brain Cognitive Development (ABCD) Study®. *Dev Cogn Neurosci* 51: 101005.

20. Brislin SJ, Martz ME, Joshi S, Duval ER, Gard A, Clark DA, *et al.* (2022): Differentiated nomological networks of internalizing, externalizing, and the general factor of psychopathology (‘p factor’) in emerging adolescence in the ABCD study. *Psychol Med* 52: 3051–3061.

fMRIPrep Details

The following was generated automatically by fMRIPrep software and is copied here unchanged:

Results included in this manuscript come from preprocessing performed using *fMRIPrep* 1.5.0 (Esteban, Markiewicz, et al. (2018); Esteban, Blair, et al. (2018); RRID:SCR_016216), which is based on *Nipype* 1.2.2 (Gorgolewski et al. (2011); Gorgolewski et al. (2018); RRID:SCR_002502).

Anatomical data preprocessing

The T1-weighted (T1w) image was corrected for intensity non-uniformity (INU) with `N4BiasFieldCorrection` (Tustison et al. 2010), distributed with ANTs 2.2.0 (Avants et al. 2008, RRID:SCR_004757), and used as T1w-reference throughout the workflow. The T1w-reference was then skull-stripped with a *Nipype* implementation of the `antsBrainExtraction.sh` workflow (from ANTs), using OASIS30ANTs as target template. Brain tissue segmentation of cerebrospinal fluid (CSF), white-matter (WM) and gray-matter (GM) was performed on the brain-extracted T1w using `fast` (FSL 5.0.9, RRID:SCR_002823, Zhang, Brady, and Smith 2001). Brain surfaces were reconstructed using `recon-all` (FreeSurfer 6.0.1, RRID:SCR_001847, Dale, Fischl, and Sereno 1999), and the brain mask estimated previously was refined with a custom variation of the method to reconcile ANTs-derived and FreeSurfer-derived segmentations of the cortical gray-matter of Mindboggle (RRID:SCR_002438, Klein et al. 2017). Volume-based spatial normalization to one standard space (MNI152NLin6Asym) was performed through nonlinear registration with `antsRegistration` (ANTs 2.2.0), using brain-extracted versions of both T1w reference and the T1w template. The following template was selected for spatial normalization: *FSL's MNI ICBM 152 non-linear 6th Generation Asymmetric Average Brain Stereotaxic Registration Model* [Evans et al. (2012), RRID:SCR_002823; TemplateFlow ID: MNI152NLin6Asym].

Functional data preprocessing

For each of the 10 BOLD runs found per subject (across all tasks and sessions), the following preprocessing was performed. First, a reference volume and its skull-stripped version were generated using a custom methodology of *fMRIPrep*. A deformation field to correct for susceptibility distortions was estimated based on two echo-planar imaging (EPI) references with opposing phase-encoding directions, using `3dQwarp` Cox and Hyde (1997) (AFNI 20160207). Based on the estimated susceptibility distortion, an unwarped BOLD reference was calculated for a more accurate co-registration with the anatomical reference. The BOLD reference was then co-registered to the T1w reference using `bbregister` (FreeSurfer) which implements boundary-based registration (Greve and Fischl 2009). Co-registration was configured with six degrees of freedom. Head-motion parameters with respect to the BOLD reference (transformation matrices, and six corresponding rotation and translation parameters) are estimated before any

spatiotemporal filtering using `mcflirt` (FSL 5.0.9, Jenkinson et al. 2002). The BOLD time-series, were resampled to surfaces on the following spaces: *fsaverage5*. The BOLD time-series (including slice-timing correction when applied) were resampled onto their original, native space by applying a single, composite transform to correct for head-motion and susceptibility distortions. These resampled BOLD time-series will be referred to as *preprocessed BOLD in original space*, or just *preprocessed BOLD*. The BOLD time-series were resampled into standard space, generating a *preprocessed BOLD run in ['MNI152NLin6Asym'] space*. First, a reference volume and its skull-stripped version were generated using a custom methodology of *fMRIPrep*. Automatic removal of motion artifacts using independent component analysis (ICA-AROMA, Pruim et al. 2015) was performed on the *preprocessed BOLD on MNI space* time-series after removal of non-steady state volumes and spatial smoothing with an isotropic, Gaussian kernel of 6mm FWHM (full-width half-maximum). Corresponding "non-aggresively" denoised runs were produced after such smoothing. Additionally, the "aggressive" noise-regressors were collected and placed in the corresponding confounds file. Several confounding time-series were calculated based on the *preprocessed BOLD*: framewise displacement (FD), DVARS and three region-wise global signals. FD and DVARS are calculated for each functional run, both using their implementations in *Nipype* (following the definitions by Power et al. 2014). The three global signals are extracted within the CSF, the WM, and the whole-brain masks. Additionally, a set of physiological regressors were extracted to allow for component-based noise correction (*CompCor*, Behzadi et al. 2007). Principal components are estimated after high-pass filtering the *preprocessed BOLD* time-series (using a discrete cosine filter with 128s cut-off) for the two *CompCor* variants: temporal (tCompCor) and anatomical (aCompCor). tCompCor components are then calculated from the top 5% variable voxels within a mask covering the subcortical regions. This subcortical mask is obtained by heavily eroding the brain mask, which ensures it does not include cortical GM regions. For aCompCor, components are calculated within the intersection of the aforementioned mask and the union of CSF and WM masks calculated in T1w space, after their projection to the native space of each functional run (using the inverse BOLD-to-T1w transformation). Components are also calculated separately within the WM and CSF masks. For each CompCor decomposition, the *k* components with the largest singular values are retained, such that the retained components' time series are sufficient to explain 50 percent of variance across the nuisance mask (CSF, WM, combined, or temporal). The remaining components are dropped from consideration. The head-motion estimates calculated in the correction step were also placed within the corresponding confounds file. The confound time series derived from head motion estimates and global signals were expanded with the inclusion of temporal derivatives and quadratic terms for each (Satterthwaite et al. 2013). Frames that exceeded a threshold of 0.5 mm FD or 1.5 standardised DVARS were annotated as motion outliers. All resamplings can be performed with *a single interpolation step* by composing all the pertinent transformations (i.e. head-motion transform matrices, susceptibility distortion correction when available, and co-registrations to anatomical and output spaces). Gridded (volumetric) resamplings were performed using `antsApplyTransforms` (ANTs), configured with Lanczos interpolation to minimize the smoothing effects of other kernels (Lanczos 1964). Non-gridded (surface) resamplings were performed using `mri_vol2surf` (FreeSurfer).

Many internal operations of *fMRIPrep* use *Nilearn* 0.5.2 (Abraham et al. 2014, RRID:SCR_001362), mostly within the functional processing workflow. For more details of the pipeline, see the section corresponding to workflows in *fMRIPrep*'s documentation.

Copyright Waiver

The above boilerplate text was automatically generated by fMRIPrep with the express intention that users should copy and paste this text into their manuscripts *unchanged*. It is released under the CC0 license.

References

Abraham, Alexandre, Fabian Pedregosa, Michael Eickenberg, Philippe Gervais, Andreas Mueller, Jean Kossaifi, Alexandre Gramfort, Bertrand Thirion, and Gael Varoquaux. 2014. "Machine Learning for Neuroimaging with Scikit-Learn." *Frontiers in Neuroinformatics* 8. https://doi.org/10.3389/fninf.2014.00014.

Avants, B.B., C.L. Epstein, M. Grossman, and J.C. Gee. 2008. "Symmetric Diffeomorphic Image Registration with Cross-Correlation: Evaluating Automated Labeling of Elderly and Neurodegenerative Brain." *Medical Image Analysis* 12 (1): 26–41. https://doi.org/10.1016/j.media.2007.06.004.

Behzadi, Yashar, Khaled Restom, Joy Liau, and Thomas T. Liu. 2007. "A Component Based Noise Correction Method (CompCor) for BOLD and Perfusion Based fMRI." *NeuroImage* 37 (1): 90–101. https://doi.org/10.1016/j.neuroimage.2007.04.042.

Cox, Robert W., and James S. Hyde. 1997. "Software Tools for Analysis and Visualization of fMRI Data." *NMR in Biomedicine* 10 (4-5): 171–78. https://doi.org/10.1002/(SICI)1099-1492(199706/08)10:4/5<171::AID-NBM453>3.0.CO;2-L.

Dale, Anders M., Bruce Fischl, and Martin I. Sereno. 1999. "Cortical Surface-Based Analysis: I. Segmentation and Surface Reconstruction." *NeuroImage* 9 (2): 179–94. https://doi.org/10.1006/nimg.1998.0395.

Esteban, Oscar, Ross Blair, Christopher J. Markiewicz, Shoshana L. Berleant, Craig Moodie, Feilong Ma, Ayse Ilkay Isik, et al. 2018. "FMRIPrep." *Software*. Zenodo. https://doi.org/10.5281/zenodo.852659.

Esteban, Oscar, Christopher Markiewicz, Ross W Blair, Craig Moodie, Ayse Ilkay Isik, Asier Erramuzpe Aliaga, James Kent, et al. 2018. "fMRIPrep: A Robust Preprocessing Pipeline for Functional MRI." *Nature Methods*. https://doi.org/10.1038/s41592-018-0235-4.

Evans, AC, AL Janke, DL Collins, and S Baillet. 2012. "Brain Templates and Atlases." *NeuroImage* 62 (2): 911–22. https://doi.org/10.1016/j.neuroimage.2012.01.024.

Gorgolewski, K., C. D. Burns, C. Madison, D. Clark, Y. O. Halchenko, M. L. Waskom, and S. Ghosh. 2011. "Nipype: A Flexible, Lightweight and Extensible Neuroimaging Data Processing Framework in Python." *Frontiers in Neuroinformatics* 5: 13. https://doi.org/10.3389/fninf.2011.00013.

Gorgolewski, Krzysztof J., Oscar Esteban, Christopher J. Markiewicz, Erik Ziegler, David Gage Ellis, Michael Philipp Notter, Dorota Jarecka, et al. 2018. "Nipype." *Software*. Zenodo. https://doi.org/10.5281/zenodo.596855.

Greve, Douglas N, and Bruce Fischl. 2009. "Accurate and Robust Brain Image Alignment Using Boundary-Based Registration." *NeuroImage* 48 (1): 63–72. https://doi.org/10.1016/j.neuroimage.2009.06.060.

Jenkinson, Mark, Peter Bannister, Michael Brady, and Stephen Smith. 2002. "Improved Optimization for the Robust and Accurate Linear Registration and Motion Correction of Brain Images." *NeuroImage* 17 (2): 825–41. https://doi.org/10.1006/nimg.2002.1132.

Klein, Arno, Satrajit S. Ghosh, Forrest S. Bao, Joachim Giard, Yrjö Häme, Eliezer Stavsky, Noah Lee, et al. 2017. "Mindboggling Morphometry of Human Brains." *PLOS Computational Biology* 13 (2): e1005350. https://doi.org/10.1371/journal.pcbi.1005350.

Lanczos, C. 1964. "Evaluation of Noisy Data." *Journal of the Society for Industrial and Applied Mathematics Series B Numerical Analysis* 1 (1): 76–85. https://doi.org/10.1137/0701007.

Power, Jonathan D., Anish Mitra, Timothy O. Laumann, Abraham Z. Snyder, Bradley L. Schlaggar, and Steven E. Petersen. 2014. "Methods to Detect, Characterize, and Remove Motion Artifact in Resting State fMRI." *NeuroImage* 84 (Supplement C): 320–41. https://doi.org/10.1016/j.neuroimage.2013.08.048.

Pruim, Raimon H. R., Maarten Mennes, Daan van Rooij, Alberto Llera, Jan K. Buitelaar, and Christian F. Beckmann. 2015. "ICA-AROMA: A Robust ICA-Based Strategy for Removing Motion Artifacts from fMRI Data." *NeuroImage* 112 (Supplement C): 267–77. https://doi.org/10.1016/j.neuroimage.2015.02.064.

Satterthwaite, Theodore D., Mark A. Elliott, Raphael T. Gerraty, Kosha Ruparel, James Loughead, Monica E. Calkins, Simon B. Eickhoff, et al. 2013. "An improved framework for confound regression and filtering for control of motion artifact in the preprocessing of resting-state functional connectivity data." *NeuroImage* 64 (1): 240–56. https://doi.org/10.1016/j.neuroimage.2012.08.052.

Tustison, N. J., B. B. Avants, P. A. Cook, Y. Zheng, A. Egan, P. A. Yushkevich, and J. C. Gee. 2010. "N4ITK: Improved N3 Bias Correction." *IEEE Transactions on Medical Imaging* 29 (6): 1310–20. https://doi.org/10.1109/TMI.2010.2046908.

Zhang, Y., M. Brady, and S. Smith. 2001. “Segmentation of Brain MR Images Through a Hidden Markov Random Field Model and the Expectation-Maximization Algorithm.” *IEEE Transactions on Medical Imaging* 20 (1): 45–57. https://doi.org/10.1109/42.906424.
